# Combining national survey with facility-based HIV testing data to obtain more accurate estimate of HIV prevalence in districts in Uganda

**DOI:** 10.1186/s12889-020-8436-z

**Published:** 2020-03-23

**Authors:** Joseph Ouma, Caroline Jeffery, Joseph J. Valadez, Rhoda K. Wanyenze, Jim Todd, Jonathan Levin

**Affiliations:** 1 Division of Epidemiology and Biostatistics, School of Public Health, University of Witwatersrand, Johannesburg, South Africa; 2 METRe Group, Department of International Health, Liverpool School of Tropical Medicine, Pembroke Place, Liverpool, L3 5QA UK; 3 Department of Disease Control and Environmental Health, Makerere University School of Public Health, Kampala, Uganda; 4 Department of Population Health, Faculty of Epidemiology and Population Health, London School of Hygiene and Tropical Medicine, London, UK

**Keywords:** Combining, Bias, Population survey, Health Information System, Hybrid Prevalence Estimate, District Health Information System

## Abstract

**Background:**

National or regional population-based HIV prevalence surveys have small sample sizes at district or sub-district levels; this leads to wide confidence intervals when estimating HIV prevalence at district level for programme monitoring and decision making. Health facility programme data, collected during service delivery is widely available, but since people self-select for HIV testing, HIV prevalence estimates based on it, is subject to selection bias. We present a statistical annealing technique, Hybrid Prevalence Estimation (HPE), that combines a small population-based survey sample with a facility-based sample to generate district level HIV prevalence estimates with associated confidence intervals.

**Methods:**

We apply the HPE methodology to combine the 2011 Uganda AIDS indicator survey with the 2011 health facility HIV testing data to obtain HIV prevalence estimates for districts in Uganda. Multilevel logistic regression was used to obtain the propensity of testing for HIV in a health facility, and the propensity to test was used to combine the population survey and health facility HIV testing data to obtain the HPEs. We assessed comparability of the HPEs and survey-based estimates using Bland Altman analysis.

**Results:**

The estimates ranged from 0.012 to 0.178 and had narrower confidence intervals compared to survey-based estimates. The average difference between HPEs and population survey estimates was 0.00 (95% CI: − 0.04, 0.04). The HPE standard errors were 28.9% (95% CI: 23.4–34.4) reduced, compared to survey-based standard errors. Overall reduction in HPE standard errors compared survey-based standard errors ranged from 5.4 to 95%.

**Conclusions:**

Facility data can be combined with population survey data to obtain more accurate HIV prevalence estimates for geographical areas with small population survey sample sizes. We recommend use of the methodology by district level managers to obtain more accurate HIV prevalence estimates to guide decision making without incurring additional data collection costs.

## Background

Accurate data are needed for monitoring health programmes and interventions and for appropriate allocation of resources. In most of sub-Saharan Africa (SSA), where the HIV/AIDS epidemic is generalized, national population surveys, such as AIDS Indicator Surveys (AIS), are preferred to provide reliable health indicator estimates for programme monitoring [1]. The surveys are however designed to provide estimates at national and regional levels, but small sample sizes at district or sub-district levels lead to less reliable indicator estimates, that have wider confidence intervals [1–4].

Health Information Systems such as the District Health Information System (DHIS2) provide another source of information that can be used for monitoring the HIV/AIDS epidemic. This data is collected more regularly, available at more decentralized levels, e.g. districts and costs less to collect. WHO, UNAIDS and other development partners recommend use of routine facility data in addition to other data sources to monitor programme performance, assess intervention coverage and measure levels of disease in a population [5]. Use of routine health facility data informed the adjustments in HIV prevalence estimates in many countries in Eastern and Southern Africa [6]. Several other studies highlight the utility of data from routine service delivery in informing service delivery decisions [7, 8]. Routine service delivery data, however, are collected only on individuals who attend/access health facilities and thus provide potentially biased estimates of population indicators.

In addition, development partners and ministries of health in middle and low-income countries have invested in electronic health information systems including the DHIS2, to improve the quality and timeliness of data from the systems. In Uganda, Ministry of Health (MoH) with support from development partners conduct quarterly reviews to validate data reported into DHIS2 [9]. With this investment, there is a need to find ways to utilize this source of information to inform service delivery decisions. Combining routine data with a relatively small sample of respondents from population survey data has been found to produce more accurate indicator estimates [10, 11].

Statistical models in packages such as SPECTRUM or THEMBISA attempt to use both routine and population survey data to calculate HIV/AIDS indicators. Model inputs such as ANC prevalence, mortality, number of individuals on ART and recent HIV prevalence when not available, complicate their use [12]. A simpler and more robust method may be easier to use and give good results.

Larmarange and Bendaud obtained district level estimates from population survey data from 17 countries using a kernel density approach implemented in PrevR [13]. In districts with inadequate number of observations in the survey sample, estimates were obtained based on observations from neighboring districts or administrative units and were categorized as “uncertain” estimates [13]. Using a similar approached, PrevR, UNAIDS found “uncertain” estimates in up to 86% of the districts in Mozambique and in 79% of the districts in Uganda [13, 14].

In this study, we explore use of the readily available health facility service delivery data in combination with population survey data to obtain more accurate HIV prevalence estimates at district level for monitoring interventions and disease impact in the general population. We implement the Hybrid Prevalence Estimation (HPE) methodology to obtain HIV prevalence estimate and 95% confidence interval for districts in Uganda. The estimation process accounts for sample size limitations associated with population survey data at district level and self-selection bias associated with health facility testers, a limitation that many researchers have not been able to address adequately [2–4, 15–18].

## Methods

### Data sources

We analyzed data from the 2011 Uganda AIDS Indicator Survey (UAIS) and health facility HIV testing data from the national DHIS2 collected during 2011. UAIS data was downloaded from the Measure DHS website www.measuredhs.com after obtaining consent from ICF/Macro international, while health facility testing data was extracted from the DHIS2 hosted at MoH after obtaining written permission from MoH. Ethical clearance to conduct this study was obtained from the University of Witwatersrand Human Research Ethics Committee (HREC) and Uganda National Council for Science and Technology (UNCST).

### Uganda AIDS Indicator survey

The UAIS is a nationally representative, population-based, HIV serological survey, designed to provide HIV prevalence estimates at national and regional levels [19]. The survey used a two-stage cluster sampling design. For the 2011 survey, Uganda was divided into 10 geographical regions each consisting of 8–15 neighboring districts. Clusters were randomly selected from each region with probability proportional to number of households in the cluster. The estimated number of households per cluster were projections from the 2002 National Population and Housing Census (NPHC) [20]. Clusters were enumeration areas from the 2002 NPHC. Sample sizes were allocated equally across the 10 geographical regions. A systematic sample of 25 households were then selected from each cluster using the 2002 NPHC sampling frame. All adults present in the selected household and who consented to participate in the survey were interviewed [19]. More details about the survey are available from www.measuredhs.com.

For this study, a total of 19,475 individuals (8532 men and 10,943 women) aged 15–49 years and tested for HIV during the survey were considered. Variables included in the analysis were (i) at cluster level: area of residence (urban/rural) and region of the country and at (ii) individual level: respondents’ gender, marital status, education level attained, number of sexual partners including husband/wife in the 12 months preceding the survey, employment status and distance to nearest health facility.

A multilevel logistic regression model was fitted to the UAIS data to obtain the respondents’ probability of testing for HIV in a health facility. The model was fitted using a total of 470 clusters. The average number of observations per cluster were 45(min = 11 and max = 64). Unequal sample selection probabilities were accounted for by incorporating scaled sampling weights. Carle’s methodology was applied to adjust/scale the sampling weights [21]. The models were fitted using maximum likelihood method in Stata statistical software, release 15 [22].

Survey respondents were considered to have tested for HIV at a health facility if they reported that they tested for HIV in health facility and received their test results in the 12 months preceding the survey. Pregnant or breastfeeding women who tested for HIV during antenatal care attendance and individuals who tested at an HIV care centre such as The AIDS Support Organization (TASO) and AIDS Information Centre (AIC) were included in the analysis. Health facilities included facilities owned and managed by government (public) and private organizations that reported HIV testing data to the national DHIS2.

### Health facility data

Health facility HIV testing data comprised of data reported to the national DHIS2. The system was developed to provide accurate, timely and quality routine data for monitoring and planning for the health sector in Uganda [9, 23]. Training and technical support from development partners and MoH has led to improvement in the quality and reliability of data in the system [9]. Aggregated HIV testing data is reported by health facilities to the DHIS2 on a monthly basis. The data includes HIV testing at all inpatient and outpatient departments in health facilities. For 2011 reporting period, data was disaggregated by age (i.e. 0–14, 15–49 and 50+ years) and gender (male and female). For this study, we considered males and females aged 15–49 years.

Indicators considered for this analysis were: number of individuals who were tested and received their HIV test results (A) and number of individuals who tested HIV positive (B). For ANC data, we considered number of pregnant women counseled, tested and received their HIV test results (C) at first antenatal visit and the number who tested HIV positive (D). HIV counseling and testing algorithm in Uganda recommends HIV testing for any individual whose most recent negative HIV test result was conducted more than 3 months prior to the current visit to the health facility [24]. Some individuals may test multiple times within a year but may not disclose to health workers resulting in double counting, a key limitation for this study. Furthermore, some pregnant women may test for HIV before seeking antenatal care and test again during antenatal attendance leading to double counting in the data reported to the national DHIS2.

Variables based on DHIS2 data were defined as follows;

- Total number of individuals tested for HIV = (*A* + *C*)
- Number HIV positive = (*B* + *D*)
- Total number of males tested for HIV = *males in A*
- Number of males tested HIV positive = males in *B*
- Total number of females tested for HIV = (*females in A*) + *C*
- Number of female tested HIV positive = (*females in B*) + *D*

### Addressing possible bias in health facility data

Routine facility data collected as part of service delivery consists of individuals who self-select, limiting its’ use for general population health indicator monitoring. To obtain general population indicator estimates, some researchers have used census projections as denominators, however this approach often results in coverage estimates that are greater than 100% [25]. Population surveys are preferred to obtain health indicator denominators since their design takes into account population distribution in the country [25–28]. The UAIS comprise two subpopulations, namely individuals who tested for HIV in a health facility in the 12 months preceding the survey (the facility testers) and those who did not test for HIV in a health facility (the non-facility testers) for the same period. We assume that the UAIS estimates of HIV prevalence for those who tested for HIV in a health facility and for those who did not test for HIV in a health facility are accurate at regional levels, since estimates of domain proportions from a multistage survey are unbiased. We apply this assumption to adjust the denominators of the DHIS2 data so that at the regional level, DHIS2 HIV prevalence estimates are similar to UAIS prevalence estimates. The adjustment process was carried out as follows:

1. We obtained the HIVs prevalence ${\hat{k}}_f$ among health facility testers in each region in the UAIS data.
2. We adjusted denominators in the DHIS2 data for each region using ${n}_{ajdusted}^r=\frac{n_{pos}}{{\hat{k}}_f}$, where *n*_*pos*_ is the observed number of individuals who tested HIV positive in each region in the DHIS2 data.
3. Calculated an adjustment factor (*δ*_*f*_) for each region, using ${\delta}_f=\frac{n_{ajdusted}^r}{n_r}$, where *n*_*r*_ is the observed number of individuals who tested for HIV in each region from the DHIS2 data.
4. We applied the adjustment factor (*δ*_*f*_), to obtain ${n}_{ajdusted}^d$, the adjusted number of individuals who tested for HIV in a health facility at district level using, ${n}_{ajdusted}^d={\delta}_f\ast {n}_d$, where *n*_*d*_ is the observed number of individuals tested for HIV at district level.
5. HIV prevalence (*P*_*f*_) based on DHIS2 adjusted data in the district was then obtained as a ratio of *n*_*pos*_, the total observed positives and *n*_*adjusted*_ the adjusted number of individuals who tested for HIV in the district, i.e. ${P}_f=\frac{n_{pos}}{n_{ajdusted}^d}$

### Hybrid prevalence estimation methodology

We consider *n* individuals in the UAIS to include *n*_*c*_ individuals who tested for HIV at a health facility during the 12 months preceding the survey and know their test result and ${n}_{\underset{\_}{c}}$ individuals who did not test for HIV at a health facility and therefore do not know their HIV status. i.e. $n={n}_c+{n}_{\underset{\_}{c}}$. Using health facility prevalence computed in step 5 above, we computed district HIV prevalence as a weighted average of prevalence from DHIS2 data, *P*_*f*_ and prevalence among individuals who did not test for HIV in a health facility, ${\hat{P}}_{\underset{\_}{s}}$ estimated from the UAIS data.

1 $$ \hat{P}={\hat{\pi}}_c{P}_f+\left(1-{\hat{\pi}}_c\right){\hat{P}}_{\underset{\_}{s}} $$

where;

$\hat{P}$ – HPE/combined estimate, ${\hat{\pi}}_c$ – the estimated probability of testing for HIV in a health facility, *P*_*f*_− Adjusted HIV prevalence for individuals tested at a health facility and ${\hat{P}}_{\underset{\_}{s}}$ – HIV prevalence for individuals tested during the survey and had not tested for HIV in a health facility in the 12 months preceding the survey. We estimated ${\hat{\pi}}_c$ from UAIS data using multilevel logistic regression adjusting for both individual and cluster level factors. Applying this model, we account for clustering at cluster level [25]. Although the probability of testing for HIV in a health facility was obtained at individual level, we used average district level probability of testing to combine the estimates. Since average probability of HIV testing is obtained from a survey sample containing both facility and non-facility testers, we estimate the variance and standard errors (SE) for the HPE respectively as follows;

2 $$ {\displaystyle \begin{array}{l} Var\left(\hat{P}\right)=\frac{1}{n}\left\{{\hat{P}}_{\underset{\_}{s}}\left(1-{\hat{P}}_{\underset{\_}{s}}\right)\left(1-{\hat{\pi}}_c\right)+\left(1-{\hat{\pi}}_c\right)\ {\left({P}_f-{\hat{P}}_{\underset{\_}{s}}\right)}^2\right\}\\ {}\mathrm{and}\kern0.37em SE=\sqrt{\mathit{\operatorname{var}}\left(\hat{P}\right)}\end{array}} $$

We assess accuracy of the HPEs compared to survey-based prevalence estimates by computing the percentage change in standard errors. We further assessed agreement of the estimates obtained using the HPE methodology with those from population survey method (Direct population survey estimate) using a Bland Altman analysis [26, 27].

All analysis was carried out in Stata statistical analysis software, Release 15 [22] and R version 3.5.0 [28].

## Results

Of the 19,475 individuals, 6729 (34.6%) tested for HIV in a health facility in the 12 months preceding the survey. HIV prevalence among those who tested in a health facility was 0.084 compared to 0.068 among those who did not test in a health facility (Table 1).

**Table 1:** Regional level HIV prevalence estimates

Region	Population Survey prevalence (in proportion) (Number HIV+)			Health Facility Prevalence (Number HIV+)^a^
	Overall	Tested in Health Facility	Not tested in health-Facility	
**Central 1**	0.106 (185)	0.123 (74)	0.096 (111)	0.094 (40,880)
**Central 2**	0.090 (166)	0.079 (57)	0.096 (109)	0.070 (36,125)
**East Central**	0.057 (108)	0.081 (39)	0.048 (69)	0.037 (17,207)
**Kampala**	0.071 (156)	0.080 (62)	0.066 (94)	0.098 (25,447)
**Mid-Eastern**	0.041 (88)	0.042 (24)	0.041 (64)	0.040 (13,926)
**North East**	0.053 (98)	0.053 (45)	0.052 (53)	0.026 (15,106)
**Mid Northern**	0.083 (159)	0.099 (81)	0.071 (78)	0.076 (39,559)
**Mid-Western**	0.083 (170)	0.089 (63)	0.080 (107)	0.062 (52,102)
**West Nile**	0.048 (88)	0.055 (33)	0.045 (55)	0.031 (7758)
**South Western**	0.080 (149)	0.103 (64)	0.069 (85)	0.053 (29,338)
**National**	0.073 (1367)	0.084 (542)	0.068 (825)	0.058 (277,448)

NOTE: Regional HIV Prevalence from population survey and health facility testing data

^a^HIV prevalence from the unadjusted health facility data

From health facility data, national (unadjusted) HIV prevalence was 0.058 (Male: 0.057 Female: 0.059). A total of 4,758,991 (female: 73.7%) individuals were tested for HIV in a health facility. (Table 1). DHIS2 HIV positivity by gender is presented Additional file 1: Appendix 1.

### Weighting/annealing factor

**Fig. 1:**
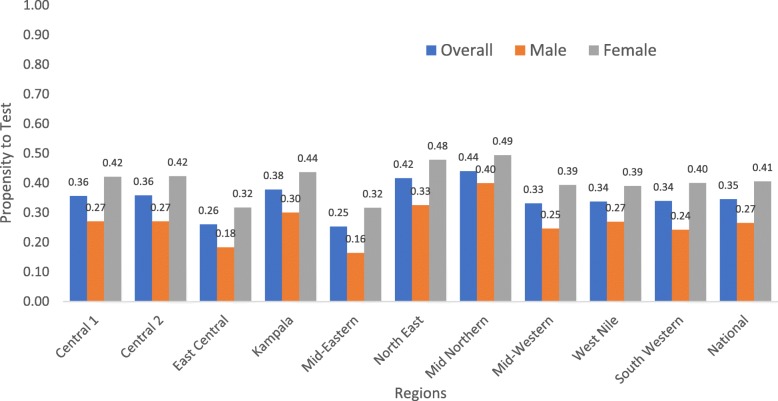
Propensity to test for HIV in a health facility

Overall (national) average propensity to test in a health facility was 0.35 (male: 0.27, female:0.41). It ranged from 0.001 to 0.95 (Fig. 1). Mid Northern region had the highest average propensity to test for HIV in health facility, 0.44 (male: 0.40 and female: 0.49) while Mid-Eastern region had the lowest, 0.25 (male: 0.16, female: 0.32) (Fig. 1).

### Hybrid prevalence estimates

HIV prevalence was highest in Central 1 region (0.11) and lowest in Mid-Western and Mid-Eastern regions (0.04 in each region). District level HPEs ranged from 0.01 to 0.18. Average HIV prevalence by region were; Central 1: 0.11 (min; 0.06, max; 0.18), Central 2: 0.10 (0.08, 0.17), East Central: 0.05 (0.02, 0.09), Mid-Eastern: 0.04 (0.01, 0.09), Mid Northern: 0.09 (0.05, 0.14), Mid-Western: 0.08 (0.03,0.16), North East:0.04 (0.04, 0.10), South West: 0.08 (0.04, 0.13) and West Nile: 0.04 (0.03, 0.07) (Table 2). Table 2 also presents HPE, survey and DHIS2 based district HIV prevalence estimates by district.

**Table 2:** HPE HIV prevalence estimates, (HPE, Survey and unadjusted DHIS2)

Region and District	HPE		Population Survey		Facility Data (unadjusted)
	Estimate (95% CI)		Estimate (95% CI)		
**Central 1**					
Bukomansimbi	0.064	(0.027, 0.101)	0.074	(0.021, 0.127)	0.083
Butambala	0.103	(0.017, 0.190)	0.124	(0.000, 1.000)	0.063
Gomba	0.090	(0.036, 0.144)	0.158	(0.060, 0.257)	0.063
Kalangala	0.138	(0.064, 0.212)	0.195	(0.000, 1.000)	0.135
Kalungu	0.142	(0.065, 0.219)	0.102	(0.025, 0.179)	0.086
Lwengo	0.143	(0.090, 0.195)	0.157	(0.090, 0.223)	0.105
Lyantonde	0.121	(0.012, 0.229)	0.109	(0.000, 1.000)	0.085
Masaka	0.178	(0.129, 0.227)	0.156	(0.096, 0.216)	0.131
Mpigi	0.097	(0.065, 0.129)	0.108	(0.059, 0.158)	0.101
Rakai	0.081	(0.053, 0.109)	0.076	(0.042, 0.109)	0.067
Sembabule	0.072	(0.037, 0.106)	0.067	(0.015, 0.118)	0.078
Wakiso	0.107	(0.090, 0.124)	0.096	(0.070, 0.123)	0.094
**Central 2**					
Buikwe	0.083	(0.060, 0.106)	0.079	(0.042, 0.116)	0.086
Buvuma	0.170	(0.102, 0.238)	0.185	(0.096, 0.273)	0.085
Kayunga	0.069	(0.041, 0.096)	0.065	(0.030, 0.100)	0.049
Kiboga	0.090	(0.035, 0.145)	0.063	(0.008, 0.117)	0.069
Kyankwanzi	0.111	(0.049, 0.173)	0.128	(0.054, 0.203)	0.045
Luwero	0.094	(0.066, 0.123)	0.080	(0.044, 0.117)	0.075
Mityana	0.136	(0.097, 0.175)	0.104	(0.058, 0.151)	0.125
Mubende	0.077	(0.053, 0.101)	0.094	(0.056, 0.132)	0.057
Mukono	0.090	(0.063, 0.116)	0.092	(0.054, 0.129)	0.069
Nakaseke	0.073	(0.038, 0.109)	0.083	(0.025, 0.141)	0.083
Nakasongola	0.083	(0.041, 0.125)	0.076	(0.018, 0.134)	0.088
**East Central**					
Bugiri	0.037	(0.019, 0.055)	0.027	(0.008, 0.045)	0.029
Buyende	0.055	(0.020, 0.091)	0.044	(0.006, 0.081)	0.033
Iganga	0.062	(0.037, 0.087)	0.054	(0.025, 0.082)	0.035
Jinja	0.088	(0.064, 0.112)	0.090	(0.056, 0.124)	0.057
Kaliro	0.036	(0.001, 0.071)	0.046	(0.000, 0.098)	0.023
Kamuli	0.051	(0.034, 0.068)	0.058	(0.033, 0.082)	0.036
Luuka	0.023	(0.005, 0.042)	0.018	(0.000, 0.037)	0.018
Mayuge	0.042	(0.023, 0.062)	0.092	(0.043, 0.140)	0.036
Namayingo	0.089	(0.043, 0.135)	0.093	(0.035, 0.151)	0.079
Namutumba	0.037	(0.011, 0.062)	0.038	(0.000, 0.076)	0.030
**Kampala**	0.076	(0.067, 0.085)	0.071	(0.058, 0.084)	0.098
**Mid Eastern**					
Budaka	0.065	(0.018, 0.111)	0.050	(0.007, 0.093)	0.029
Bududa	0.025	(0.000, 0.055)	0.046	(0.006, 0.087)	0.014
Bulambuli	0.026	(0.000, 0.055)	0.023	(0.000, 0.053)	0.040
Busia	0.068	(0.038, 0.097)	0.074	(0.037, 0.112)	0.063
Butaleja	0.026	(0.003, 0.049)	0.026	(0.001, 0.052)	0.018
Kapchorwa	0.092	(0.000, 0.186)	0.097	(0.000, 1.000)	0.021
Kibuku	0.036	(0.000, 0.074)	0.014	(0.000, 0.040)	0.027
Kween	0.013	(0.000, 0.032)	0.007	(0.000, 0.022)	0.022
Manafwa	0.034	(0.014, 0.055)	0.034	(0.006, 0.062)	0.035
Mbale	0.037	(0.022, 0.052)	0.035	(0.015, 0.055)	0.070
Pallisa	0.012	(0.002, 0.022)	0.007	(0.000, 0.019)	0.021
Sironko	0.051	(0.022, 0.079)	0.050	(0.020, 0.080)	0.030
Tororo	0.071	(0.044, 0.097)	0.069	(0.041, 0.098)	0.044
**Mid Northern**					
Agago	0.080	(0.047, 0.113)	0.046	(0.011, 0.081)	0.071
Alebtong	0.066	(0.035, 0.097)	0.058	(0.016, 0.100)	0.068
Amolatar	0.108	(0.058, 0.159)	0.158	(0.079, 0.237)	0.078
Amuru	0.059	(0.027, 0.091)	0.028	(0.000, 0.061)	0.043
Apac	0.062	(0.043, 0.080)	0.052	(0.022, 0.082)	0.081
Dokolo	0.070	(0.041, 0.099)	0.055	(0.015, 0.094)	0.061
Gulu	0.090	(0.066, 0.113)	0.100	(0.049, 0.151)	0.086
Kitgum	0.081	(0.042, 0.121)	0.083	(0.024, 0.141)	0.072
Kole	0.047	(0.023, 0.070)	0.029	(0.001, 0.057)	0.051
Lamwo	0.088	(0.041, 0.134)	0.121	(0.043, 0.198)	0.071
Lira	0.108	(0.079, 0.138)	0.111	(0.066, 0.157)	0.098
Nwoya	0.123	(0.033, 0.212)	0.163	(0.000, 1.000)	0.052
Otuke	0.136	(0.048, 0.224)	0.128	(0.000, 1.000)	0.075
Oyam	0.066	(0.043, 0.089)	0.068	(0.035, 0.100)	0.058
Pader	0.140	(0.088, 0.192)	0.165	(0.090, 0.239)	0.087
**Mid Western**					
Buliisa	0.065	(0.013, 0.117)	0.038	(0.000, 0.785)	0.065
Bundibugyo	0.032	(0.010, 0.054)	0.032	(0.000, 0.071)	0.028
Hoima	0.075	(0.050, 0.100)	0.086	(0.051, 0.121)	0.053
Kabarole	0.159	(0.124, 0.194)	0.137	(0.094, 0.180)	0.099
Kamwenge	0.065	(0.037, 0.093)	0.054	(0.023, 0.086)	0.050
Kasese	0.066	(0.046, 0.085)	0.050	(0.027, 0.073)	0.050
Kibaale	0.068	(0.045, 0.091)	0.079	(0.046, 0.113)	0.049
Kiryandongo	0.059	(0.019, 0.098)	0.071	(0.016, 0.126)	0.048
Kyegegwa	0.118	(0.058, 0.177)	0.127	(0.056, 0.198)	0.046
Kyenjojo	0.077	(0.047, 0.106)	0.121	(0.071, 0.172)	0.070
Masindi	0.063	(0.035, 0.091)	0.060	(0.023, 0.097)	0.062
**North East**					
Abim	0.066	(0.004, 0.128)	0.098	(0.000, 1.000)	0.031
Amudat	0.090	(0.000, 0.204)	0.049	(0.000, 0.899)	0.022
Amuria	0.102	(0.067, 0.138)	0.102	(0.056, 0.148)	0.039
Bukedea	0.044	(0.014, 0.074)	0.045	(0.002, 0.088)	0.022
Kaabong	0.037	(0.016, 0.057)	0.021	(0.000, 0.044)	0.022
Kaberamaido	0.081	(0.045, 0.117)	0.061	(0.020, 0.102)	0.034
Katakwi	0.074	(0.041, 0.107)	0.080	(0.034, 0.126)	0.035
Kotido	0.052	(0.009, 0.095)	0.046	(0.000, 0.096)	0.017
Kumi	0.040	(0.023, 0.057)	0.025	(0.000, 0.051)	0.024
Moroto	0.041	(0.014, 0.069)	0.065	(0.000, 0.132)	0.023
Nakapiripirit	0.049	(0.002, 0.097)	0.038	(0.000, 0.792)	0.018
Napak	0.075	(0.018, 0.132)	0.073	(0.003, 0.143)	0.023
Ngora	0.046	(0.015, 0.078)	0.061	(0.014, 0.108)	0.020
Serere	0.049	(0.029, 0.069)	0.046	(0.014, 0.077)	0.028
Soroti	0.089	(0.060, 0.118)	0.058	(0.019, 0.097)	0.034
**South Western**					
Buhweju	0.035	(0.000, 0.077)	0.022	(0.000, 0.592)	0.025
Bushenyi	0.096	(0.060, 0.132)	0.079	(0.036, 0.123)	0.068
Ibanda	0.065	(0.027, 0.102)	0.053	(0.002, 0.104)	0.059
Isingiro	0.102	(0.061, 0.143)	0.112	(0.064, 0.160)	0.045
Kabale	0.043	(0.024, 0.063)	0.037	(0.010, 0.064)	0.035
Kanungu	0.077	(0.042, 0.112)	0.084	(0.036, 0.131)	0.047
Kiruhura	0.095	(0.046, 0.144)	0.086	(0.025, 0.148)	0.072
Kisoro	0.053	(0.015, 0.091)	0.059	(0.012, 0.105)	0.026
Mbarara	0.101	(0.068, 0.134)	0.124	(0.072, 0.177)	0.059
Mitooma	0.097	(0.042, 0.151)	0.076	(0.012, 0.140)	0.069
Ntungamo	0.063	(0.040, 0.085)	0.062	(0.030, 0.095)	0.050
Rubirizi	0.133	(0.067, 0.199)	0.140	(0.044, 0.236)	0.057
Rukungiri	0.082	(0.053, 0.111)	0.076	(0.038, 0.115)	0.064
Sheema	0.095	(0.056, 0.134)	0.124	(0.063, 0.185)	0.069
**West Nile**					
Adjumani	0.044	(0.020, 0.067)	0.039	(0.012, 0.065)	0.023
Arua	0.043	(0.031, 0.056)	0.049	(0.030, 0.069)	0.035
Koboko	0.048	(0.019, 0.077)	0.063	(0.025, 0.102)	0.025
Maracha	0.029	(0.001, 0.058)	0.007	(0.000, 0.019)	0.014
Moyo	0.033	(0.011, 0.055)	0.037	(0.005, 0.069)	0.019
Nebbi	0.068	(0.045, 0.091)	0.070	(0.042, 0.098)	0.036
Yumbe	0.026	(0.010, 0.042)	0.027	(0.006, 0.048)	0.014
Zombo	0.056	(0.031, 0.082)	0.040	(0.011, 0.069)	0.050

**Fig. 2:**
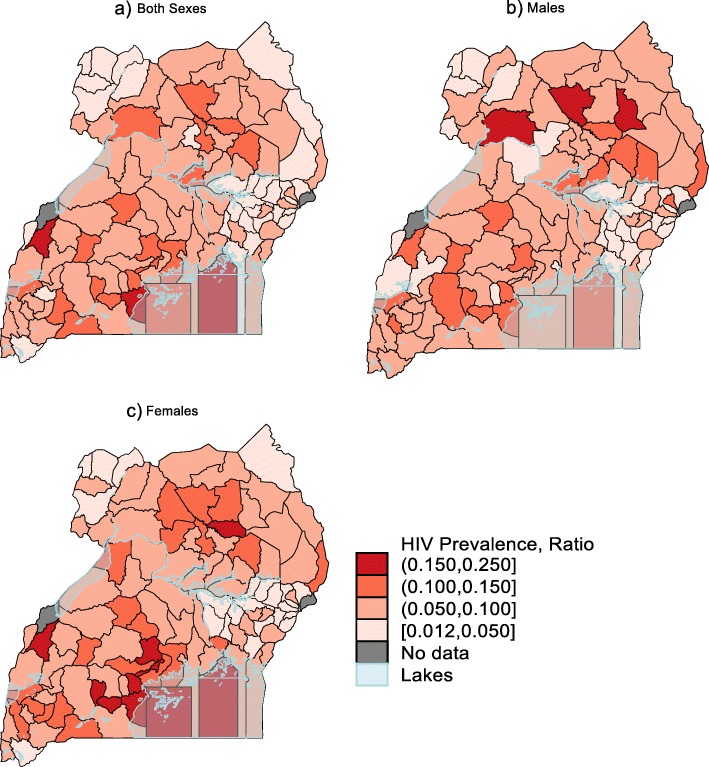
District Hybrid Prevalence Estimates. Maps created based on study data using Stata Statistical Software: Release 15. User licence was acquired before using the software

Figure 2 presents HIV prevalence maps in; both sexes (map a); in males (map b); and in females (map c). HPEs had similar patterns for both sexes, males and females consistent with the regional level prevalence estimates from population survey in Table 1. Districts in Central 1 region, Mid northern region, Island, and those along lake shores had higher overall, male and female HIV prevalence estimates (Fig. 2, and Additional file 1: Appendix 2) while districts in mid-eastern and West Nile region had lower HIV prevalence estimates. HPEs were not calculated for two districts (Bukwo in mid-eastern region and Ntoroko in mid-western region) because UAIS data points was not available for those districts.

**Fig. 3:**
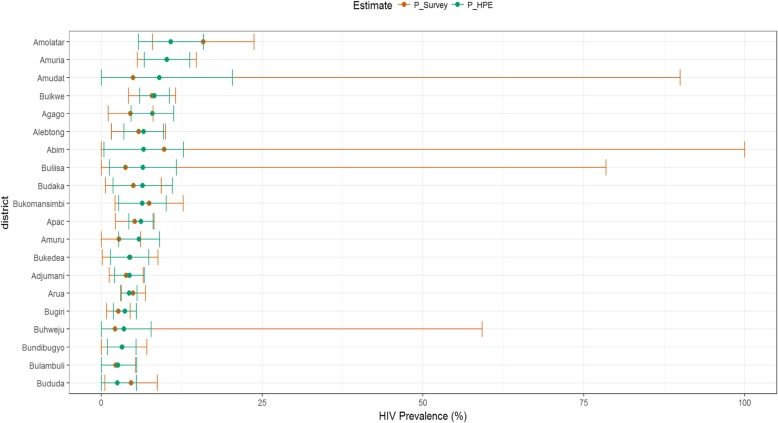
District prevalence estimates from combined and population survey data. P_survey- survey based prevalence estimate while P_HPE is HIV prevalence based on the HPE methodology

**Fig. 4:**
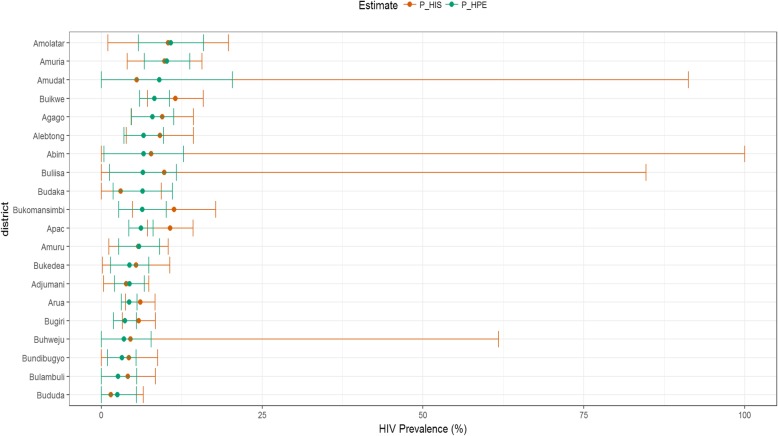
District prevalence estimates from combined and DHIS2 data. P_HIS- Health facility-based prevalence estimate while P_HPE is HIV prevalence based on the HPE methodology

Figure 3 compares district HIV prevalence estimates from population survey and HPE while in Fig. 4, we compare HPE and the adjusted DHIS2 data for selected districts. Prevalence comparison between HPE and survey for all districts is presented in Additional file 1: Appendix 3. The figures show that HPEs had narrower confidence intervals compared to direct survey estimates indicating an improvement in the precision of the estimates.

Of the 110 districts, 51 (46.4%) had lower HPEs (point estimates) and 59 (53.6%) had higher HPE compared to the survey-based district prevalence estimates (Fig. 3, Additional file 1: Appendix 3).

HPEs were however lower than the DHIS2 prevalence estimates in 74 (67.3%) and higher in 36 (32.7%) of the districts in Uganda (Fig. 4, Additional file 1: Appendix 4).

A joint comparison of the HP estimates with both survey-based and health facility-based prevalence estimates show that 33 (30.0%) of the districts had lower HPEs while 18 (16.4%) had higher estimates compared to both the survey and health facility-based prevalence estimates.

### Precision of HPE and population survey estimates

**Fig. 5:**
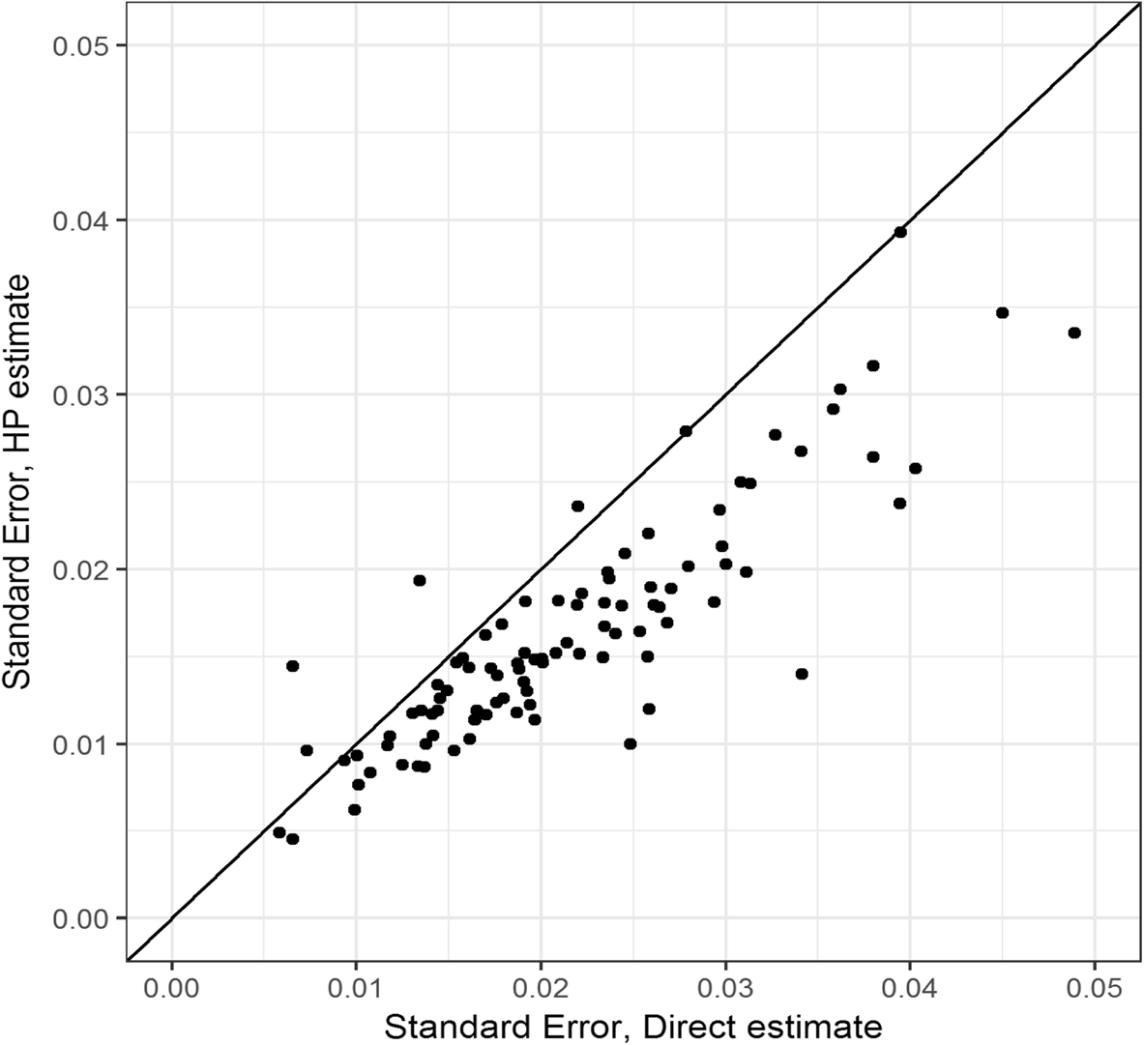
Standard errors of estimates from survey and the HPE

Standard errors of the HPEs were generally lower compared to SEs from survey-based estimates (Fig. 5). Of the districts, 105 (95.5%) had lower HPE SEs compared to SEs from survey-based estimates. Overall, the HPE standard errors were decreased by 28.9% (95% CI: 23.4–34.4) compared to survey-based standard errors.

### Similarity of HPE and survey-based estimates

**Fig. 6:**
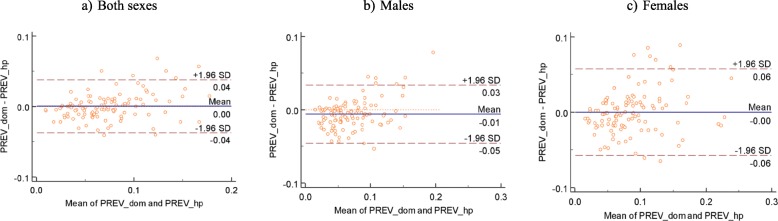
Difference plot comparing HPE and Direct survey estimates. PREV_HIS- HIV Prevalence based on health facility data, PREV_hp-HIV prevalence based on the HPE methodology while PREV_dom- HIV prevalence based on survey data only

On average, there is no difference between survey and HPE estimates, 0.0 (95% CI: − 0.04,0.04) (Fig. 6a). Average difference for males was − 0.01 (95% CI: − 0.05,0.03) while for females was 0.00 (95% CI: − 0.06,0.06). Although there seems to be a bias (0.01) when assessing the agreement between HP and survey-based estimates for males (Fig. 6b), the 95% confidence interval of the difference between the estimates are narrow. Additionally, there was no systematic pattern of the points as the average of the estimates increases.

**Fig. 7:**
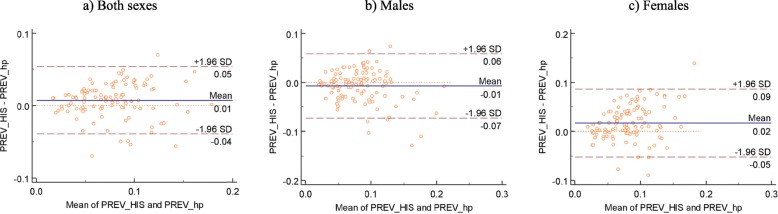
Difference plot comparing HPE and DHIS2 estimates. PREV_HIS- HIV Prevalence based on health facility data, PREV_hp-HIV prevalence based on the HPE methodology while PREV_dom- HIV prevalence based on survey data only

The mean difference between the HPE and DHIS2 estimates was 0.01 (95% CI: − 0.05,0.06) (Fig. 7a). Average difference for males was − 0.01 (95% CI: − 0.07, 0.06) while for females was 0.02 (95% CI: − 0.05, 0.09), (Fig. 7b and c respectively). The size of the difference increased with increase in the mean of the estimates. This is seen from the wider variability of the points about the no-difference (zero) line as the values of the average of the estimates increase (Fig. 7a-c). The average difference was 0.02 and confidence intervals were wider when comparing HPEs and survey-based estimates for females (Fig. 7c).

## Discussion

In this study, we implement a novel approach, the Hybrid Prevalence Estimation methodology to obtain HIV prevalence estimates for districts in Uganda. We combined DHIS2 HIV testing data with information of non-facility testers from the 2011 UAIS data to obtain district level HIV prevalence estimates.

Although national population surveys are the gold standard for calculating population level health indicators, district level estimates from these surveys are less accurate due to the reduced sample sizes at district or lower administrative levels. The demand for accurate indicator estimates at district or lower administrative levels for programme monitoring motivates use of innovative approaches to provide the estimates. We obtained district level HIV prevalence estimates by combining population survey information with DHIS2 data using a Hybrid Prevalence Estimation methodology. Our estimates had narrower confidence intervals compared to estimates from the population survey at the district level, consistent with findings elsewhere [10, 11]. The HPE was calculated from three parameters; 1) Prevalence in the health facility sample, 2) prevalence among non-facility testers from the population survey sample and 3) the propensity to test for HIV in a health facility from the population survey sample. We also observed that HIV prevalence estimate obtained using the HPE methodology was similar to the population survey HIV prevalence estimates for male and females combined, and for males only while it was lower for females. Additionally, UAIS based prevalence estimates were generally higher while DHIS2 prevalence estimates were lower than the consistent with findings elsewhere [29].

In the UAIS, the population can be divided into two domains: 1) those that have access to health facilities, get tested for HIV, and are linked to appropriate care if found HIV positive, and 2) those that do not access health facilities and may remain unknown in the health care system. Barriers to health care access for the latter subpopulation may include factors such as low/no education and not being in stable sexual relationship that also increase the risk of HIV transmission [30, 31]. Combining survey with DHIS2 data therefore generates more precise indicator estimates that can be used to improve planning and service delivery for the general population at district levels where service delivery decision are implemented.

Facility level data has known limitations including selection bias, as it is not a random sample from the population for measuring general population level prevalence [15, 16, 18, 32]. Studies in Uganda [33, 34], Tanzania [35] and Zambia [36, 37], have also found facility-based antenatal HIV testing data has biased estimates of HIV prevalence’ and therefore not appropriate for calculating HIV/AIDS indicators including HIV prevalence in the general population. The HPE methodology requires use of a small population survey sample [10, 11] to correct for bias in indicator estimates from health facility testing data. We used Uganda AIDS indicator survey data to correct for the bias in DHIS2 so as to obtain the HIV prevalence estimates for districts in Uganda. Other population surveys such as the demographic and health surveys can be similarly combined with facility-based data to obtain general population indicator estimates for planning and decision making, especially in low resourced environments where resource constraint limits collection of large sample sizes.

We applied a weighting factor, propensity to test in a health facility, calculated using multilevel logistic regression to combine the two data sources. Individuals and cluster level predictors of testing for HIV were included in the model. Predictors of access to testing or health care system may also impact HIV disease risk as noted elsewhere [10]. Multilevel logistic regression is also appropriate for the UAIS design and enables inclusion of both individual and cluster level risk factors in the modelling process. The model also accounts for clustering [21, 25, 38].

There was no difference in prevalence estimates from the HPE and Survey based approaches but confidence intervals of the HPEs were narrower, demonstrating efficiency of the HPE methodology in obtaining population level estimates as observed elsewhere [11, 18, 39].

### Strengths and limitations

We applied multilevel modeling which has multiple advantages over classical models including use of HIV risk factors at individual and cluster levels. We used data from the 2011 UAIS, a more recent survey, the Population HIV Impact Assessment (PHIA), completed in 2017 was not publicly available at the time of this study. DHS data are prone to refusal to participate, this may have bias on the results of this study as those who refuse to participate may have characteristics different from those who participated in the study. Furthermore, this study was limited to complete case analysis thus reducing the effective sample size used for the analysis. DHIS2 data includes individuals who may have tested multiple times which can lead to the use of wrong or unrepresentative denominators for individuals tested at health facilities. Studies elsewhere report repeat testing ranging from 3 to 13% [40, 41]. We further note that some health facilities, especially privately owned, do not report their data to the national DHIS2 further lowering the representativeness of health facility HIV testing data.

## Conclusions

The growing demand for accurate information for programme management and policy formulation will require strategies that use all the available information efficiently with little or no additional resource investments. Countries and development partners continue to build and strengthen DHIS2 through capacity building and regular data quality assessments. We applied a simple tool, HPE methodology, to support efficient use of DHIS data in combination with small survey samples to obtain more accurate indicator estimates at district or lower administrative levels. HPE obtained in this study had reduced standard errors (by 28.8%) compared to survey-based estimates demonstrating improved accuracy and reliability of the estimates. We therefore recommend use of the methodology to combine DHIS2 data with population survey data to obtain population level indicator estimates for lower administrative levels where the survey samples are small for accurate indicator estimation.

## Supplementary information

**Additional file 1: Appendix 1.** Population survey and DHIS2 prevalence estimates. **Appendix 2.** HPE HIV prevalence estimates and associated 95% CI. **Appendix 3.** Comparison of district prevalence estimates for the HP and survey-based estimates. **Appendix 4.** Comparison of district prevalence estimates for the HPE and DHIS2-based estimates.

## Data Availability

The 2011 AIDS indicator survey datasets analyzed during the current study are available from https://dhsprogram.com/what-we-do/survey/survey-display-373.cfm. Health facility HIV testing dataset can be accessed from Ministry of Health, Uganda following a reasonable request. Ethics clearance from Uganda National Council for Science and Technology (UNCST) is required to access the data. All data used in this study were identified by participant unique IDs, no additional identifying information was included in the data.

## Abbreviations

AIC: AIDS Information Centre
AIDS: Acquired Immune Deficiency Syndrome
AIS: Aids Indicator Survey
CI: Confidence Interval
DHIS2: District Health Information System Version 2
DHS: Demographic Health Survey
HIV: Human Immunodeficiency Virus
HPE: Hybrid Prevalence Estimate
HREC: Human Research Ethics Committee
MOH: Ministry of Health
NPHC: National Population and Housing Census
PHIA: Population HIV Impact Assessment
SE: Standard error
SSA: Sub-Saharan Africa
TASO: The AIDS Support Organization
UAIS: Uganda AIDS Indicator Survey
UNAIDS: The Joint United Nations Program on HIV/AIDS
UNCST: Uganda National Council for Science and Technology
WHO: World Health Organization
