# Supplementary material for: Combining national survey with facility-based HIV testing data to obtain more accurate estimate of HIV prevalence in districts in Uganda
Source: BMC Public Health. 2020 Mar 23;20:379. doi: 10.1186/s12889-020-8436-z (PMC7092592; doi:10.1186/s12889-020-8436-z)

**Appendix 1: Population survey and HIS prevalence estimates**

| **Region** | **Population Survey prevalence (Number HIV+)** | | | | | | **Health Facility Prevalence ( Number HIV+)** | | |
| --- | --- | --- | --- | --- | --- | --- | --- | --- | --- |
|  | Tested in Health Facility | | | Not tested in health-Facility | | |  | | |
|  | Male | Female | Male | | Female | Male | | Female |  |
| **Central 1** | 0.132 (13) | 0.120 (61) | 0.074 (50) | | 0.130 (61) | 0.094 (11,772) | | 0.095 (29,108) |  |
| **Central 2** | 0.048 (6) | 0.086 (51) | 0.086 (55) | | 0.110 (54) | 0.070 (9,308) | | 0.071 (26,817) |  |
| **East Central** | 0.055 (5) | 0.087 (34) | 0.047 (37) | | 0.049 (32) | 0.080 (5,927) | | 0.115 (19,520) |  |
| **Kampala** | 0.048 (7) | 0.088 (55) | 0.040 (37) | | 0.103 (57) | 0.044 (5,074) | | 0.035 (12,133) |  |
| **Mid-Eastern** | 0.066 (6) | 0.038 (18) | 0.036 (31) | | 0.048 (33) | 0.032 (2,695) | | 0.043 (11,231) |  |
| **North East** | 0.097 (12) | 0.043 (33) | 0.042 (25) | | 0.068 (28) | 0.026 (3,962) | | 0.026 (11,144) |  |
| **Mid Northern** | 0.088 (25) | 0.104 (56) | 0.053 (35) | | 0.097 (43) | 0.039 (2,389) | | 0.029 (5,369) |  |
| **Mid-Western** | 0.088 (11) | 0.090 (52) | 0.069 (55) | | 0.094 (52) | 0.072 (9,880) | | 0.077 (29,679) |  |
| **West Nile** | 0.072 (8) | 0.051 (25) | 0.045 (31) | | 0.044 (24) | 0.054 (8,513) | | 0.053 (20,825) |  |
| **South Western** | 0.111 (11) | 0.102 (53) | 0.059 (39) | | 0.080 (46) | 0.064 (13,712) | | 0.060 (38,390) |  |
| **National** | 0.085 (104) | 0.084 (438) | 0.057 (395) | | 0.082 (430) | 0.057 (73,232) | | 0.059 (204,216) |  |

**Appendix 2: HPE HIV prevalence estimates and associated 95% CI**

| **Region and**  **District** | **Male** | | **Female** | |
| --- | --- | --- | --- | --- |
|  | Estimate | 95% CI | Estimate | (95%CI) |
| Central 1 |  |  |  |  |
| **Bukomansimbi** | 0.036 | (0.000, 0.078) | 0.087 | (0.028, 0.145) |
| **Butambala** | 0.079 | (0.000, 0.179) | 0.164 | (0.000, 0.364) |
| **Gomba** | 0.074 | (0.010, 0.137) | 0.116 | (0.022, 0.209) |
| **Kalangala** | 0.087 | (0.012, 0.162) | 0.216 | (0.072, 0.359) |
| **Kalungu** | 0.127 | (0.024, 0.231) | 0.163 | (0.047, 0.280) |
| **Lwengo** | 0.124 | (0.051, 0.197) | 0.163 | (0.088, 0.237) |
| **Lyantonde** | 0.136 | (0.000, 0.309) | 0.232 | (0.035, 0.429) |
| **Masaka** | 0.132 | (0.070, 0.194) | 0.232 | (0.151, 0.312) |
| **Mpigi** | 0.073 | (0.038, 0.108) | 0.127 | (0.072, 0.182) |
| **Rakai** | 0.072 | (0.032, 0.113) | 0.090 | (0.049, 0.131) |
| **Sembabule** | 0.060 | (0.008, 0.112) | 0.080 | (0.032, 0.128) |
| **Wakiso** | 0.098 | (0.073, 0.122) | 0.114 | (0.089, 0.138) |
| **Central 2** |  |  |  |  |
| **Buikwe** | 0.071 | (0.041, 0.101) | 0.096 | (0.059, 0.132) |
| **Buvuma** | 0.140 | (0.060, 0.221) | 0.227 | (0.110, 0.345) |
| **Kayunga** | 0.069 | (0.029, 0.109) | 0.071 | (0.033, 0.109) |
| **Kiboga** | 0.060 | (0.009, 0.111) | 0.137 | (0.032, 0.241) |
| **Kyankwanzi** | 0.105 | (0.027, 0.182) | 0.135 | (0.020, 0.250) |
| **Luwero** | 0.098 | (0.050, 0.146) | 0.092 | (0.056, 0.127) |
| **Mityana** | 0.113 | (0.070, 0.155) | 0.183 | (0.113, 0.253) |
| **Mubende** | 0.083 | (0.046, 0.120) | 0.070 | (0.039, 0.101) |
| **Mukono** | 0.095 | (0.053, 0.137) | 0.085 | (0.051, 0.119) |
| **Nakaseke** | 0.073 | (0.017, 0.130) | 0.074 | (0.027, 0.121) |
| **Nakasongola** | 0.091 | (0.018, 0.164) | 0.075 | (0.028, 0.122) |
| **East Central** |  |  |  |  |
| **Bugiri** | 0.029 | (0.005, 0.053) | 0.044 | (0.017, 0.071) |
| **Buyende** | 0.088 | (0.015, 0.161) | 0.028 | (0.000, 0.057) |
| **Iganga** | 0.037 | (0.008, 0.066) | 0.087 | (0.046, 0.128) |
| **Jinja** | 0.072 | (0.038, 0.105) | 0.102 | (0.066, 0.137) |
| **Kaliro** | 0.031 | (0.000, 0.081) | 0.040 | (0.000, 0.090) |
| **Kamuli** | 0.059 | (0.030, 0.087) | 0.042 | (0.023, 0.061) |
| **Luuka** | 0.026 | (0.000, 0.056) | 0.020 | (0.000, 0.041) |
| **Mayuge** | 0.030 | (0.004, 0.057) | 0.052 | (0.023, 0.081) |
| **Namayingo** | 0.081 | (0.008, 0.153) | 0.093 | (0.032, 0.153) |
| **Namutumba** | 0.047 | (0.000, 0.100) | 0.059 | (0.010, 0.107) |
| **Kampala** | 0.049 | (0.038, 0.060) | 0.099 | (0.085, 0.114) |
| Mid Eastern |  |  |  |  |
| **Budaka** | 0.063 | (0.000, 0.130) | 0.070 | (0.004, 0.135) |
| **Bududa** | 0.041 | (0.000, 0.095) | 0.048 | (0.000, 0.111) |
| **Bulambuli** | 0.044 | (0.000, 0.101) | 0.048 | (0.000, 0.106) |
| **Busia** | 0.043 | (0.019, 0.068) | 0.095 | (0.045, 0.145) |
| **Butaleja** | 0.029 | (0.000, 0.062) | 0.023 | (0.000, 0.056) |
| **Kapchorwa** | 0.105 | (0.000, 0.239) | 0.077 | (0.000, 0.211) |
| **Kibuku** | 0.054 | (0.000, 0.116) | 0.074 | (0.000, 0.168) |
| **Kween** | 0.037 | (0.000, 0.088) | 0.024 | (0.000, 0.063) |
| **Manafwa** | 0.021 | (0.000, 0.045) | 0.046 | (0.013, 0.079) |
| **Mbale** | 0.027 | (0.006, 0.048) | 0.044 | (0.022, 0.066) |
| **Pallisa** | 0.015 | (0.000, 0.033) | 0.028 | (0.000, 0.056) |
| **Sironko** | 0.067 | (0.023, 0.111) | 0.031 | (0.000, 0.064) |
| **Tororo** | 0.076 | (0.040, 0.113) | 0.073 | (0.032, 0.113) |
| Mid Northern |  |  |  |  |
| **Agago** | 0.055 | (0.029, 0.082) | 0.120 | (0.048, 0.191) |
| **Alebtong** | 0.069 | (0.028, 0.110) | 0.069 | (0.022, 0.115) |
| **Amolatar** | 0.135 | (0.056, 0.215) | 0.078 | (0.024, 0.133) |
| **Amuru** | 0.054 | (0.014, 0.094) | 0.064 | (0.014, 0.115) |
| **Apac** | 0.056 | (0.031, 0.081) | 0.068 | (0.040, 0.096) |
| **Dokolo** | 0.051 | (0.023, 0.080) | 0.089 | (0.043, 0.136) |
| **Gulu** | 0.062 | (0.039, 0.085) | 0.120 | (0.079, 0.161) |
| **Kitgum** | 0.076 | (0.020, 0.133) | 0.084 | (0.030, 0.139) |
| **Kole** | 0.054 | (0.014, 0.094) | 0.067 | (0.024, 0.109) |
| **Lamwo** | 0.090 | (0.013, 0.168) | 0.079 | (0.029, 0.129) |
| **Lira** | 0.094 | (0.058, 0.130) | 0.131 | (0.081, 0.181) |
| **Nwoya** | 0.157 | (0.009, 0.305) | 0.088 | (0.001, 0.175) |
| **Otuke** | 0.125 | (0.019, 0.232) | 0.164 | (0.013, 0.316) |
| **Oyam** | 0.032 | (0.018, 0.046) | 0.115 | (0.064, 0.166) |
| **Pader** | 0.158 | (0.080, 0.236) | 0.122 | (0.058, 0.186) |
| Mid Western |  |  |  |  |
| **Buliisa** | 0.086 | (0.000, 0.187) | 0.102 | (0.010, 0.193) |
| **Bundibugyo** | 0.037 | (0.000, 0.077) | 0.057 | (0.007, 0.107) |
| **Hoima** | 0.072 | (0.037, 0.106) | 0.077 | (0.040, 0.114) |
| **Kabarole** | 0.140 | (0.093, 0.187) | 0.181 | (0.126, 0.237) |
| **Kamwenge** | 0.046 | (0.011, 0.082) | 0.081 | (0.038, 0.125) |
| **Kasese** | 0.044 | (0.019, 0.070) | 0.082 | (0.053, 0.112) |
| **Kibaale** | 0.055 | (0.023, 0.086) | 0.080 | (0.047, 0.113) |
| **Kiryandongo** | 0.045 | (0.000, 0.094) | 0.073 | (0.012, 0.134) |
| **Kyegegwa** | 0.123 | (0.038, 0.208) | 0.114 | (0.027, 0.202) |
| **Kyenjojo** | 0.080 | (0.033, 0.127) | 0.069 | (0.034, 0.104) |
| **Masindi** | 0.066 | (0.025, 0.107) | 0.058 | (0.020, 0.096) |
| North East |  |  |  |  |
| **Abim** | 0.158 | (0.000, 0.319) | 0.089 | (0.000, 0.198) |
| **Amudat** | 0.139 | (0.000, 0.334) | 0.148 | (0.000, 0.381) |
| **Amuria** | 0.101 | (0.055, 0.146) | 0.118 | (0.062, 0.174) |
| **Bukedea** | 0.063 | (0.020, 0.107) | 0.041 | (0.000, 0.085) |
| **Kaabong** | 0.062 | (0.011, 0.113) | 0.043 | (0.011, 0.076) |
| **Kaberamaido** | 0.111 | (0.064, 0.157) | 0.075 | (0.021, 0.130) |
| **Katakwi** | 0.102 | (0.052, 0.153) | 0.063 | (0.020, 0.105) |
| **Kotido** | 0.094 | (0.008, 0.181) | 0.069 | (0.000, 0.147) |
| **Kumi** | 0.042 | (0.023, 0.061) | 0.045 | (0.015, 0.076) |
| **Moroto** | 0.084 | (0.013, 0.155) | 0.059 | (0.000, 0.120) |
| **Nakapiripirit** | 0.098 | (0.000, 0.215) | 0.065 | (0.000, 0.138) |
| **Napak** | 0.086 | (0.000, 0.186) | 0.072 | (0.001, 0.144) |
| **Ngora** | 0.057 | (0.009, 0.105) | 0.039 | (0.000, 0.082) |
| **Serere** | 0.035 | (0.014, 0.055) | 0.062 | (0.025, 0.099) |
| **Soroti** | 0.089 | (0.050, 0.127) | 0.097 | (0.051, 0.143) |
| South Western |  |  |  |  |
| **Buhweju** | 0.072 | (0.000, 0.161) | 0.076 | (0.000, 0.190) |
| **Bushenyi** | 0.063 | (0.019, 0.107) | 0.124 | (0.066, 0.181) |
| **Ibanda** | 0.055 | (0.000, 0.111) | 0.072 | (0.019, 0.124) |
| **Isingiro** | 0.081 | (0.025, 0.138) | 0.122 | (0.061, 0.183) |
| **Kabale** | 0.053 | (0.016, 0.090) | 0.035 | (0.016, 0.054) |
| **Kanungu** | 0.061 | (0.021, 0.101) | 0.098 | (0.039, 0.157) |
| **Kiruhura** | 0.124 | (0.034, 0.214) | 0.066 | (0.021, 0.111) |
| **Kisoro** | 0.056 | (0.000, 0.117) | 0.052 | (0.001, 0.102) |
| **Mbarara** | 0.091 | (0.042, 0.140) | 0.110 | (0.065, 0.155) |
| **Mitooma** | 0.108 | (0.003, 0.213) | 0.088 | (0.026, 0.151) |
| **Ntungamo** | 0.057 | (0.021, 0.093) | 0.066 | (0.037, 0.095) |
| **Rubirizi** | 0.131 | (0.034, 0.228) | 0.135 | (0.045, 0.224) |
| **Rukungiri** | 0.058 | (0.025, 0.091) | 0.104 | (0.057, 0.150) |
| **Sheema** | 0.072 | (0.020, 0.125) | 0.116 | (0.058, 0.174) |
| West Nile |  |  |  |  |
| **Adjumani** | 0.033 | (0.002, 0.064) | 0.068 | (0.029, 0.106) |
| **Arua** | 0.051 | (0.031, 0.072) | 0.037 | (0.023, 0.052) |
| **Koboko** | 0.049 | (0.009, 0.089) | 0.044 | (0.004, 0.085) |
| **Maracha** | 0.042 | (0.000, 0.095) | 0.051 | (0.000, 0.105) |
| **Moyo** | 0.052 | (0.007, 0.096) | 0.038 | (0.004, 0.071) |
| **Nebbi** | 0.087 | (0.050, 0.124) | 0.050 | (0.024, 0.077) |
| **Yumbe** | 0.030 | (0.006, 0.054) | 0.021 | (0.001, 0.041) |
| **Zombo** | 0.029 | (0.007, 0.050) | 0.086 | (0.037, 0.135) |

**Appendix 3: Comparison of district prevalence estimates for the HP and survey-based estimates**


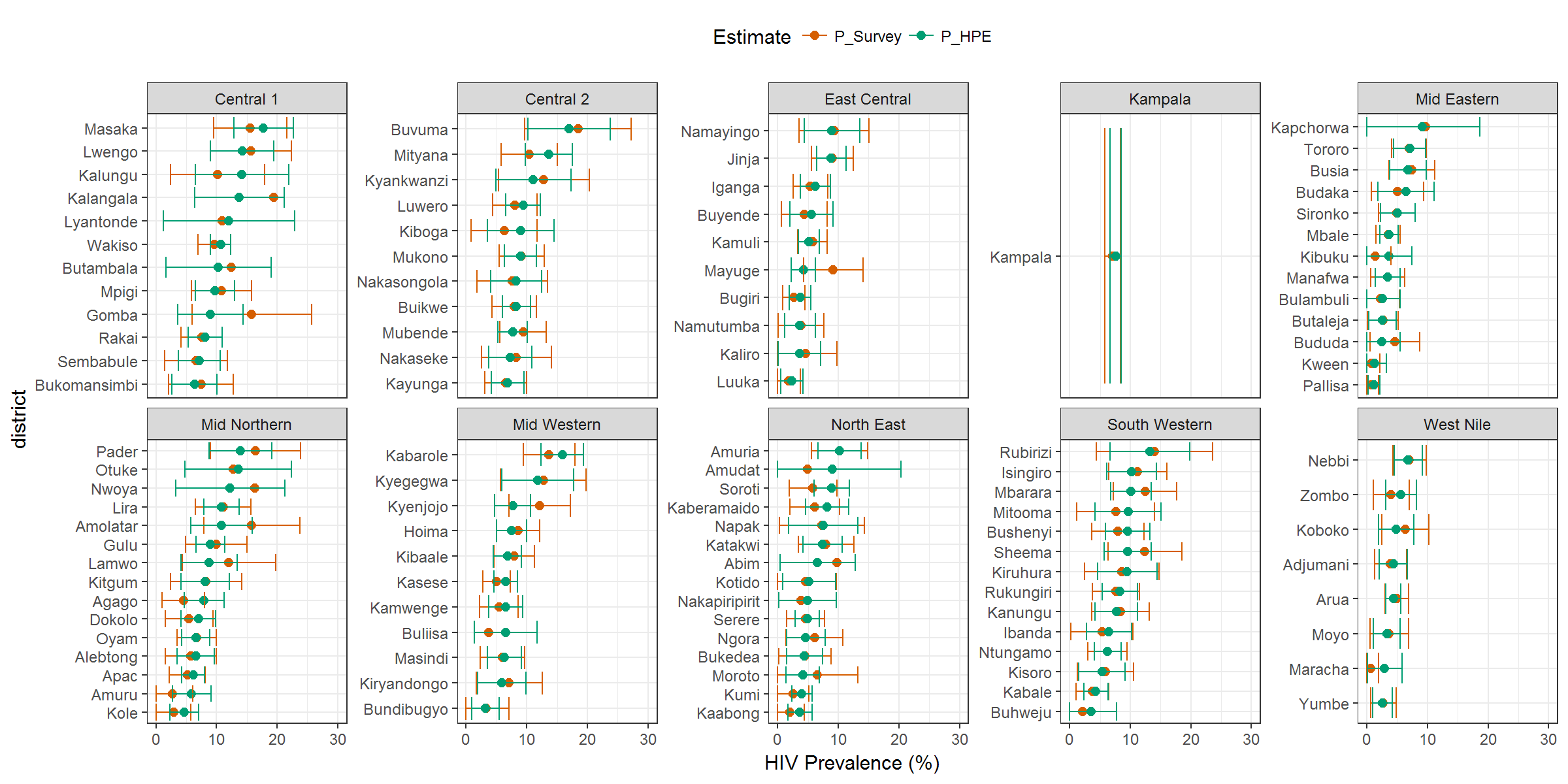


**Appendix 4: Comparison of district prevalence estimates for the HP and DHIS-based estimates**


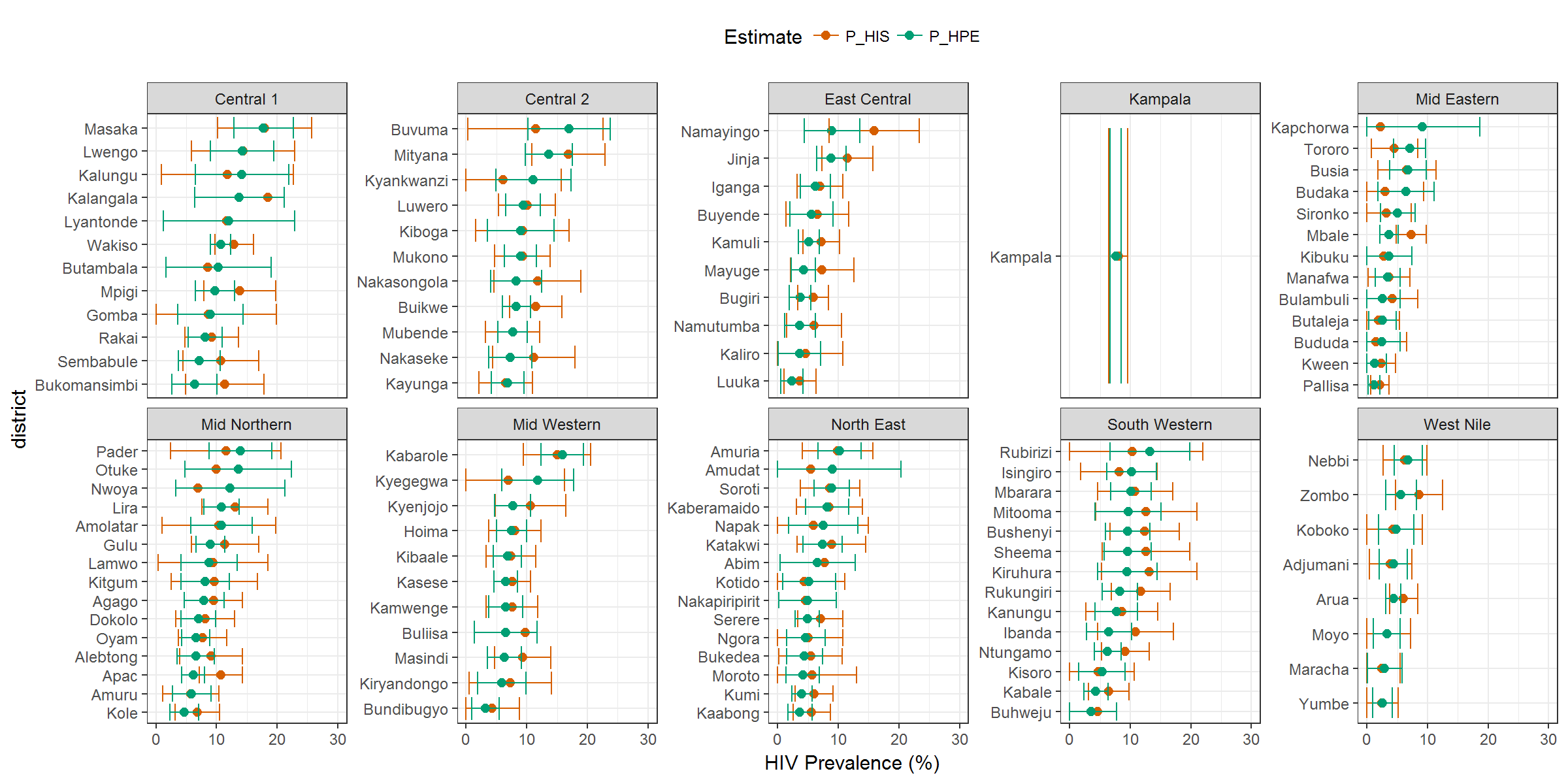

Supplement: Supplementary file 1 — Additional file 1: Appendix 1. Population survey and DHIS2 prevalence estimates. Appendix 2. HPE HIV prevalence estimates and associated 95% CI. Appendix 3. Comparison of district prevalence estimates for the HP and survey-based estimates. Appendix 4. Comparison of district prevalence estimates for the HPE and DHIS2-based estimates. [file 12889_2020_8436_MOESM1_ESM.docx]
